# Affective Colormap Design for Accurate Visual Comprehension in Industrial Tomography †

**DOI:** 10.3390/s21144766

**Published:** 2021-07-12

**Authors:** Yuchong Zhang, Morten Fjeld, Marco Fratarcangeli, Alan Said, Shengdong Zhao

**Affiliations:** 1 Department of Computer Science and Engineering, Chalmers University of Technology, SE-41296 Gothenburg, Sweden; fjeld@chalmers.se (M.F.); marcof@chalmers.se (M.F.); 2 Department of Applied Information Technology, University of Gothenburg, SE-40530 Gothenburg, Sweden; alan.said@ait.gu.se; 3 Department of Computer Science, National University of Singapore, Singapore 119077, Singapore; zhaosd@comp.nus.edu.sg

**Keywords:** affective colormap design, human perception, visualization, crowdsourced study, microwave tomography

## Abstract

The design of colormaps can help tomography operators obtain accurate visual comprehension, thereby assisting safety-critical decisions. The research presented here is about deploying colormaps that promote the best affective responses for industrial microwave tomography (MWT). To answer the two research questions related to our study, we firstly conducted a quantitative analysis of 11 frequently-used colormaps on a segmentation task. Secondly, we presented the same colormaps within a crowdsourced study comprising two parts to verify the quantitative outcomes. The first part encoded affective responses from participants into a prevailing four-quadrant valence–arousal grid; the second part recorded participant ratings towards the accuracy of each colormap on MWT segmentation. We concluded that three colormaps are the best suited in the context of MWT tasks. We also found that the colormaps triggering emotions in the positive–exciting quadrant can facilitate more accurate visual comprehension than other affect-related quadrants. A synthetic colormap design guideline was consequently proposed.

## 1. Introduction

**Figure 1:**
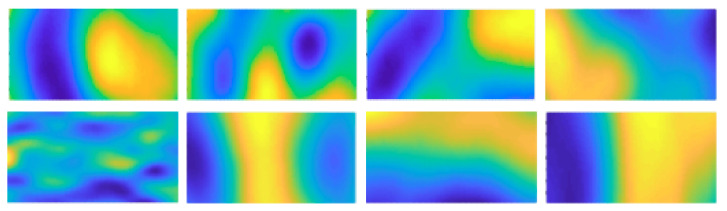
The eight MWT image samples in our study [8]. Different colors represent different foam moisture levels. Blue is the desired color, representing lower moisture levels.

Tomography is a widely-used imaging technique in medical and industrial contexts. Microwave tomography (MWT), a breed of industrial tomography, is a specific and representative tomographic technique with non-ionizing properties. MWT is commonly used in industrial process applications [1]; it can significantly contribute to a more sustainable process by reducing energy and material needs. A critical problem in ensuring such benefits is an accurate control of the heating process. There is a specific application through MWT called microwave drying [2] for porous foams [3]. The significant parameter, moisture level, is deployed to characterize the success of the whole industrial process. In this context, MWT images, offering information that can be visualized using colormaps, are central in controlling the heating process. An operator’s visual comprehension of an MWT image is key in recognizing the moisture levels on images. Figure 1 shows the set of eight MWT image samples used in our study. Each sample was acquired from a confined microwave foam drying process and reveals post-process moisture levels. Different colors represent different moisture levels regarding the post-dried foam involved in this process. For example, blue parts signify low moisture levels.

Appropriate color scheme usage in graphs, images and animations can enhance expressiveness and persuasiveness in visual representations. The goal of color-mapping is to effectively communicate these features from visual imagery to those data that are the most prominent in hands-on tasks [4]. Color is a retinal variable which is conventionally determined by hue, saturation and brightness (HSB); all three being dimensions in perception-based applications [5]. These three perceptual dimensions combined with different choices and values, cause the diversification of colormaps. Information visualizations aim to seek the most suitable representation using visual features to support cognitive data interpretation [6]. Research has proven that using different colormaps can result in differing interpretations, depending on how the visualization is perceived by the human eye [7], that is, the selection of colormaps can significantly influence a user’s visual comprehension of data.

In addition to the visual imagery, people also react emotionally to different colormaps. Emotions can influence how the information presented to people will be interpreted and how people will be affected in the visual environment [9]. The psychology of color has demonstrated a tight correlation between specific colors and human affect, which could lead to different applications in various fields. Affect plays a role in information visualization, including communicative intention, engagement and storytelling [10,11]. A successful deployment of colormaps can not only improve the objective performance of tasks but can also arouse affective resonance, as well as raising visual immersion. For instance, an image with a gloomy colormap may trigger a sense of depression while the one with a bright color scheme may incite gladness. Since MWT is an image-driven methodology with the need of human comprehension, the appropriate choice of colormaps sparking distinct affect is considered in our research.

**Figure 2:**
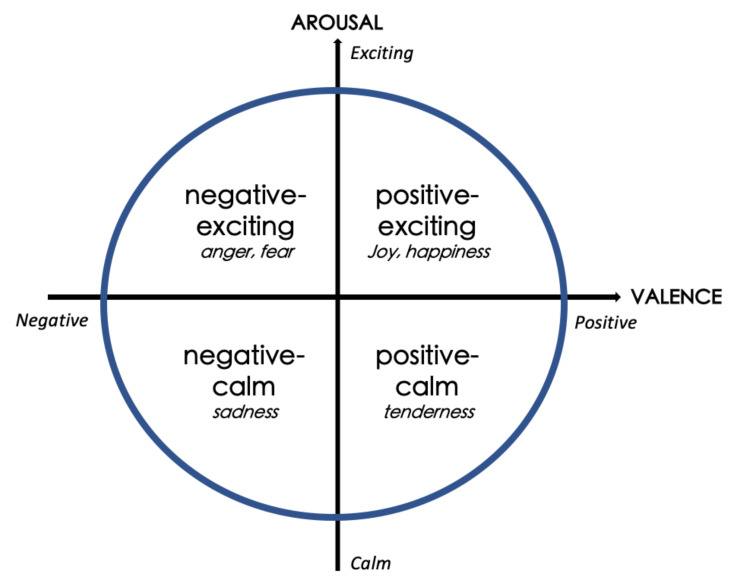
The circumplex valence–arousal affective model used in our study [12]. We hypothesize that the **positive–exciting** quadrant is more desirable than other quadrants.

To measure the effect of different colormaps explored within our study, we firstly propose a research question. **RQ1**: How can various colormaps affect domain related task accuracy in the context of MWT so as to support accurate visual comprehension? Besides, we note that research has shown how colors can be linked to affective expressions. A commonly-used approach to determine affect is to encode it with the circumplex valence–arousal emotional model [6,12,13,14,15] for further analysis. Emotions are characterized and plotted as a 2D circumplex coordinate system graph (Figure 2) with the first dimension being **positive** and **negative** (valence) and the second dimension being physically **exciting** and **calm** (arousal) [13]. These psychological dimensions of affect are significantly influenced by informative properties of colormaps, such as lightness and chroma. For example, the **calm** affect can be triggered by using light, cool and pastel colormaps [6]. Some design rules for colormaps have been developed for practical uses, suggesting that the chromatic properties of colormaps are emphasized and increased in **positive** and **exciting** status and are weakened and reduced in **negative** and **calm** status [14]. This leads to the second research question. **RQ2**: Are colormaps triggering affect in the **positive–exciting** quadrant of the valence–arousal grid able to facilitate more accurate visual comprehension in terms of human perception towards MWT than those from the other three quadrants? According to Bartram et al. [6], more strongly saturated colors can be used to characterize the affect **positive** quadrant, while **positive** emphasizes higher chroma colors. Since the segmentation task, which is highly dependent on deeper colors is engaged in our study, we hypothesize that the **positive–exciting** quadrant is more desirable than other quadrants.

In this paper, we concentrate on colormap design for visual comprehension of industrial tomography, featuring MWT images based on a segmentation task. To balance energy effectiveness, material flow and safety aspects, it is crucial that humans accurately interpret such images. To resolve **RQ1**, we implemented a systematic quantitative study focusing on an MWT image segmentation task to evaluate the colormaps. To tackle **RQ2** and validate our hypothesis, we formulated and conducted a crowdsourced study on the same task. In our previous published work [8], we demonstrated how we conducted the quantitative evaluation to evaluate the colormaps in the context of MWT. Thus, as the full extension of that work [8], the main contributions of this paper are as follows:

- Investigating how different colormaps affect task accuracy in the context of MWT by a quantitative evaluation and obtaining the colormaps yielding the best accuracy.
- Combining conventional design study with a crowdsourced study and validating that colormaps triggering affect in the **positive–exciting** quadrant in the valence–arousal model are able to facilitate more precise visual comprehension in MWT.
- Proposing a synthetic design guideline for relevant researchers and practitioners to select colormaps boosting accurate visual comprehension in the context of MWT.

## 2. Related Work

In the following sub-sections, first, we focus on the state-of-the-art in colormap design, then, on the relationship between color and affect (affective colormap). Finally, we draw on these two related areas to establish the motivation of our research.

### 2.1. Colormap Design

Colormap design and selection have received attention over recent decades. In the early 1990s, Bergman et al. explored a rule-based tool to help choose the best colormap for isomorphic, segmentation and highlighting tasks [16]. Schulze-Wollgast et al. exploited an enhanced automatic color-coding framework by encapsulating metadata extraction, colormap adaptation and color legend creation [5]. Tominski et al. developed a color-coding function to choose color scales according to particular tasks [17]. Similarly, Mittelstädt et al. [18] proposed a guided tool for selecting suitable colormaps for combined analysis tasks. It is noteworthy that Schloss et al. [7] associated colormaps with people’s reasoning towards color-quantity mappings. For example, they report such inferred color-mappings: dark-is-more and opaque-is-more biases. By conducting several hands-on crowdsourcing experiments with appropriate participants, Reda et al. [19] designed a guideline which indicates that the rainbow scheme or diverging colormaps afford superior accuracy for tasks requiring gradient perception. Likewise, Turton et al. [20] also leveraged a crowdsourced tool called Ware color key to assess various colormaps.

### 2.2. Affective Colormap

There is a body of research that has been focusing on the intersection between colors, affect, cognition and behavior [21,22]. Wilms et al. [23] state that the effect of a certain color on emotions depends not on only a single property but on a combination, such as hue, saturation and brightness. In a similar fashion, Bartram et al. [6] concluded that colormaps have affective expression. They inspected the relationship between affect and color properties (hue, chroma and lightness) and confirmed the most advisable palette composition design principles with regard to the corresponding emotions. Some affective color-mapping rules were developed by Yang et al. [14] to encode visual properties of colormaps with the valence–arousal model for re-rendering in animation. In practical application settings, the valence–arousal emotion model is widely adopted due to its functionality. Kragel et al. [24] utilized this model to embody complex affect on humans and have constructed a comprehensive emotion-estimating framework in visual systems.

### 2.3. Motivation

Previous work has successfully proven that the usage of appropriate color coding plays a critical role in related data or tasks. In numerous cases, researchers generate colormap design principles for better data visualization by comparative studies through specific tasks [5,16,25]. Furthermore, integrating color-mapping and affect has been a ubiquitous research topic in the domain of affective color-coding. People combine empirical assessment with miscellaneous user studies to examine the link between colormaps and human affect, enhancing the information visualization [6,14,26]. In our research, we particularly deploy a combined study consisting of an objective quantitative assessment with a subjective crowdsourced study for the domain of industrial tomography. The novelty of our study is that by applying the affective colormap design guidelines derived through an automatic segmentation task, researchers and practitioners may obtain an up-to-date tool to support both visualization and visual comprehension in the domain of industrial tomography.

## 3. Methodology

### 3.1. Overview

The MWT images included in this study were obtained from eight different and independent industrial microwave foam drying processes, as shown in Figure 1. These reconstructed images possess an intrinsic continuous colormap when being handled in MATLAB, which is denoted as *parula*. Our objectives were to design desirable colormaps yielding superior task accuracy for those MWT images and to test whether the colormaps eliciting affect in the **positive–exciting** part of the valence–arousal model were able to boost visual comprehension. Therefore, we adopted an image segmentation task to visualize the preferred blue parts (in colormap *parula*, as shown in Figure 1), corresponding to low moisture levels of the used foam in original MWT images.

**Figure 3:**
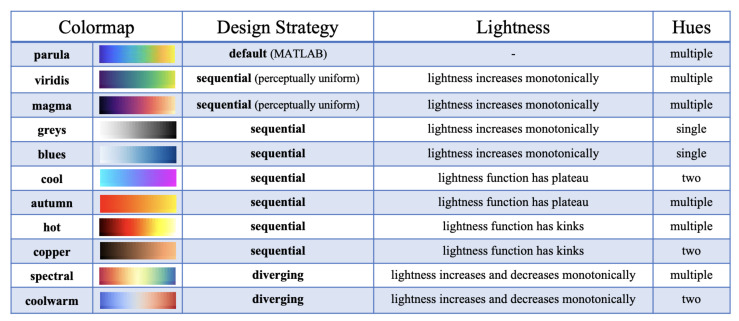
The 11 colormaps we studied with their hues and lightness characteristics, followed by each colormap’s underlying design strategy [8].

First, after conducting a literature review, we chose another 10 commonly-used continuous colormaps (listed and elaborated in Figure 3 (the order of the colormaps presented keeps consistent through the paper)). To answer **RQ1**, we implemented a quantitative evaluation to assess the performance of colormaps in MWT through the segmentation task by deploying the same quantitative assessment as proposed in [8]. **RQ2**, as the main part of this study, was explored by involving a systematic crowdsourced user study which is detailed in Section 4.

### 3.2. Colormaps

Our aim was to investigate whether the commonly adopted colormaps, which vary in their degrees of lightness and hues and differ in their efficiency and effectiveness in the same segmentation task. The following selection of colormaps is presented based on recently published colormap design papers and follows five (4 + 1) design strategies [8].

– **Sequential:** Change in lightness and often incremental saturation of color, often using a single hue, should be used for representing information that has hierarchy.
  * **Sequential 1:** Perceptually uniform, with each new color equally perceptually distinct from the previous and following colors.
  * **Sequential 2:** Monotonical increase of lightness values.
  * **Sequential 3:** In the lightness function space, there is a plateau, or the function may go both up and down.
  * **Sequential 4:** In the lightness function space, there are some kinks in the function.
– **Diverging:** Change in lightness and possibly saturation of two different colors that meet in the middle at an unsaturated color; used in information being plotted that has a critical middle value, such as topography or when data deviate around zero.
  For each colormap, a specific design strategy is elaborated as follows.
  * **parula:** The default colormap in MATLAB.
  * **viridis:** The default blue-green-yellow colormap in Matplotlib (the plotting library for the Python programming language); a popular sequential colormap [25,26,27].
  * **magma:** Another perceptually-uniform black-purple-pink colormap [25,26,27].
  * **greys:** Simple grayscale color bar [7,26].
  * **blues:** Simple blue color bar [7,19,26].
  * **cool:** Cyan-magenta color map; based on a colormap of the same name in MATLAB [16].
  * **autumn:** Sequential increasing shades of red-orange-yellow [7].
  * **hot:** Sequential black-red-yellow-white, to emulate blackbody radiation from an object at increasing temperatures [7].
  * **copper:** Sequential increasing shades of black-copper [25].
  * **spectral:** Diverging, multi-hue encompassing a subset of the rainbow with a yellow middle [19].
  * **coolwarm:** Diverging blue-gray-red, meant to avoid issues with 3D shading, color blindness and ordering of colors [19,27].

### 3.3. Quantitative Evaluation for Colormaps

**Figure 4:**
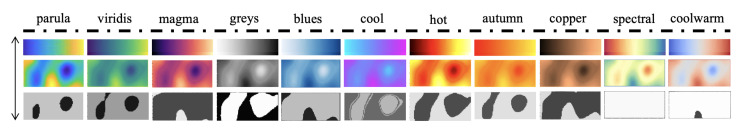
Top-down image processing pipeline (arrow): Each of the 11 colormaps (1st row) is applied to the same MWT image resulting in a new image (2nd row) and yielding corresponding segmented images (3rd row). Due to limited space, we randomly chose one MWT image from our total of eight. The goal of segmentation was to visualize the blue parts in the colormap *parula* [8].

After selection, we converted our eight MWT images with the 11 chosen colormaps (obtaining a total of 88 MWT images) by using OpenCV (Open Source Computer Vision Library, referred as a library comprising various programming functions aiming for real-time computer vision: https://opencv.org, accessed on 18 April 2020). Thus, we were able to observe each colormap in segmenting the desired low moisture areas (blue parts on the images in *parula* colormap). With this implementation, we established the underlying quality of the selected 11 colormaps in the context of the MWT segmentation task. Due to the limited space and for better interpretation, we randomly chose one image shown in Figure 1 (the second image in the first row from left) for exemplification (Figure 4). We then executed the same segmentation among all 88 MWT images. Different colormaps with distinct color properties (hue, lightness, saturation, etc.) were liable to result in divergent outcomes in segmentation tasks [16]. The segmentation of each image was conducted by an automatic method MWTS-KM [28,29] containing three key steps: image augmentation, grayscale conversion and k-means implementation. The automatic nature of this method constructed the link between the colormaps and the segmentation task we carried out. After initial observations (Figure 4), we first inferred that *parula*, *viridis*, *cool*, *hot* and *autumn* schemes are capable of visualizing the blue parts on the source image in segmentation. However, a systematic quantitative evaluation was then followed by adopting a data-driven approach [8,29], where three indexes (Jaccard index, Dice coefficient and false positive, as illustrated in Equations (1)–(3), with Source denoting the source MWT image to be segmented while Segmentation represents the segmented image) were used to compare segmented and corresponding ground-truth images presented in the respective 11 colormaps. The explanation of these three indicators is narrated as follows [29]:

(1) $$\mathrm{Jaccardindex}=\frac{\left|Source\cap Segmentation\right|}{\left|Source\cup Segmentation\right|}$$

(2) $$\mathrm{Dice}\mathrm{coefficient}=2\times \frac{\left|Source|\cap |Segmentation\right|}{\left|Source|+|Segmentation\right|}$$

(3) False
positive=|Segmentation|−|Source∩Segmentation||Source|

- *Jaccard index*: The metric is used to characterize the similarity and diversity sample sets. Here, it displays the pixel-level similarity of the original and the segmented MWT images.
- *Dice coefficient*: This is another measure to obtain the similarity between two samples. It differentiates from Jaccard index in that it only calculates true positives once.
- *False positive*: This index is the proportion between the number of negative objects falsely classified as positive and the total number of negative objects. Lower value corresponds to higher accuracy.

**Figure 5:**
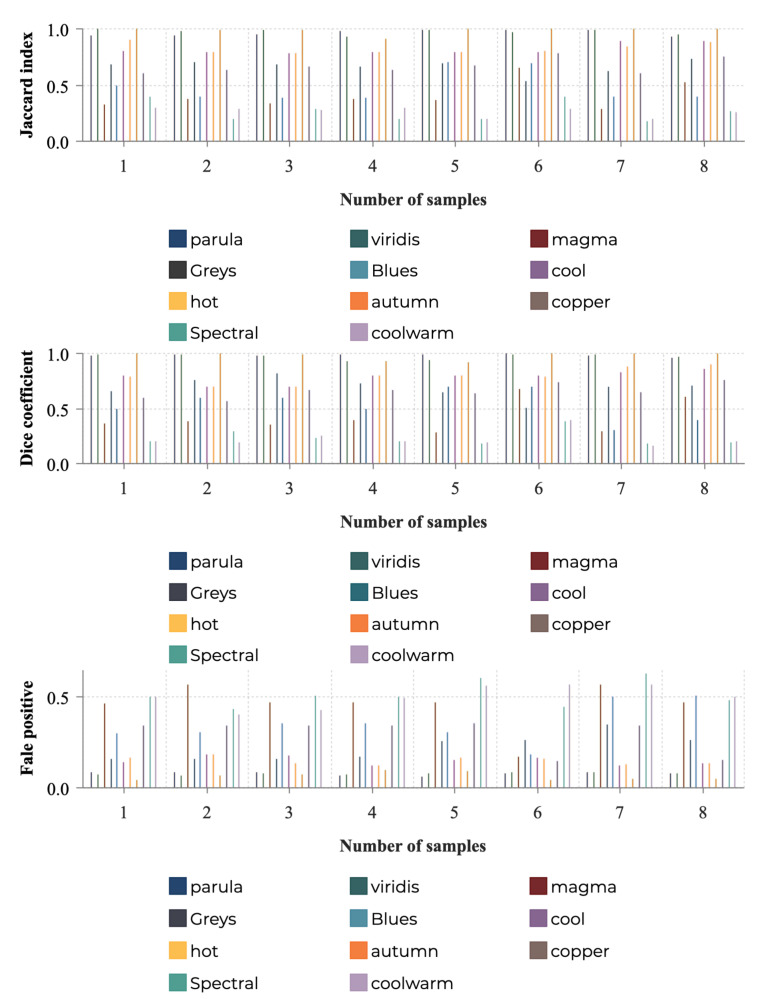
The quantitative evaluation of the 11 colormaps over 8 samples [8]. The first subfigure: Jaccard index (the higher value, the better performance); Middle subfigure: Dice coefficient (the higher value, the better performance); Third subfigure: false positive (the lower value, the better performance).

By following this procedure, we obtained the quantitative results as shown in Figure 5. After combining our initial observations and the data-driven quantitative evaluation, we concluded that *autumn*, *viridis* and *parula* schemes appear to be the most desirable choices, yielding the best domain related task accuracy in MWT, while *spectral*, *coolwarm* and *magma* schemes are much less preferable.

## 4. Crowdsourced User Study

### 4.1. Experimental Design

According to our hypothesis and **RQ2**, we intended to test whether those colormaps triggering affect in the **positive–exciting** quadrant can facilitate more accurate visual comprehension towards MWT than that from the other three quadrants in valence–arousal coordinate system. We designed a crowdsourced user study to verify our speculation. This experiment had two goals, first to examine all the participants’ affective responses to the 11 selected colormaps. The emotions from participants were encoded with this circumplex model for further analysis. Second, the user study would test participants’ comprehension ability in comparing and rating the task accuracy of diverse colormaps from the segmentation task in the prior quantitative evaluation. Hence, our study was divided into two parts. The specification of the user study is shown in Table 1, containing the stimuli presented to participants and the anticipated results from them.

The well-known valence–arousal model is an extensively used circumplex affective model [12]. In this coordinate system, valence varies from **positive** (happiness, pleasure, gladness) to **negative** (frustration, anger, distress, fear), while arousal ranges from **exciting** (high arousal, excitement, astonishment) to **calm** (sleepiness, tiredness, boredom) [6,12]. As seen in Figure 2, the common emotions are encoded and mapped in this 2D space: joy and happiness in the first quadrant (**positive–exciting**), anger and fear in the second quadrant. Sadness and tenderness are placed in the third and fourth quadrants, respectively [13].

### 4.2. Participants

Following the procedure recommended by Bigham et al. [30], “crowdsourcing broadens from amateur microtasks to goals involving groups of interdependent experts”, we constructed a class of microtasks (MWT image segmentation) with the potential to be solved by non-experts [31]. This aligns with the work of industrial process operators, for instance, operators of microwave heated oven systems. Typically, such oven operators are non-experts ensuring that the process runs smoothly (personal communication, April, 2018). Motivated by the effectiveness of crowdsourcing in scientific tomographic image analysis [31], we designed a systematic crowdsourced user study to answer our research questions. We recruited 73 participants (39 males, 53.4%; 34 females, 46.6%; mean of age = 27.5; SD of age = 2.5) based on the authors’ academic and industrial networks. From our investigation, none of these participants reported any form of color blindness so as to provide convincing and reliable data in our cognitive study. We distinguished skilled from non-experts by examining whether they had or had not been in contact with image segmentation or computer vision in the past. The skilled (*n* = 39, 53.4%) and non-experts (n = 34, 46.6%) were identified according to their prior experience in related realms.

### 4.3. Procedure: Parts I and II

We created an online questionnaire comprising two parts (I and II) to execute the crowdsourced study via Google Forms. The participants were requested to complete the study individually with no time limit. Answers were anynomized for privacy. With no color-blind participants, all results were deemed valid.

**Figure 6:**
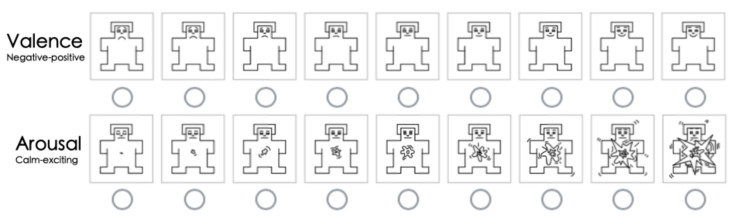
The manikins of the 9-point SAM scale used in our study.

- Part I: Participants were requested to assess their emotional responses towards the 11 colormaps, with each colormap presented in eight images derived from the original eight MWT images shown in Figure 1. We adopted the 9-point (1 to 9) Self-Assessment-Manikin (SAM) scales developed by Lang [32] to encode the affective responses of each participant. The SAM model has successfully proved its efficacy in emotional analysis [33,34,35]. To better concretize the tiers of the affect, we used 9-point SAM scales (Figure 6) for the measurement instead of invoking 5-point and 7-point scales [13]. The manikins represented different emotions as well as recording the corresponding points. In our 9-point scaling structure, participants could choose from 1 to 9 by assessing their own affect, where the point 5 was annotated as a watershed to encode affective neutrality. In the valence dimension, participants indicated their responses from negative to positive as they reacted from calm to exciting in the arousal dimension. The order of the 11 colormaps displayed in this study was randomized. Totally, 11 trials on affective assessment were conducted by each participant.
- Part II: 11 paired images were prepared, with each pair consisting of one MWT image presented in a certain colormap and one segmented image derived from the corresponding MWT image. The automatic MWTS-KM method was used for implementing each segmentation task. Likewise, the presentation order of each pair of images as well as the colormaps, were randomized to avoid order effects and enhance the convincibility. There were both accurate and inaccurate segmentation results included over these colormaps. Participants were guided to conduct the rating by comparing the segmentation results of each colormap with the foremost ground-truth–the desired low moisture areas which are the blue parts on the MWT images in *parula* colormap. Hence, in this part, participants performed the perceptual estimation task by rating the segmentation accuracy to evaluate the 11 test colormaps. Each of them followed the Likert scale [36] from 1 to 5 (very low accuracy to very high accuracy) for quantifying the rated accuracy. Throughout this procedure, participants were able to evaluate those colormaps based on their own visual comprehension. Similarly, an overall of 11 ratings were accomplished by each participant in this part.

## 5. Experimental Results

We now present the results from our crowdsourced study. We first share the results from Part I, which are the affective responses to our tested colormaps. We then display the synthetic accuracy of those colormaps rated in Part II. An additional validation analysis for our hypothesis, formulated in the introduction, is then presented.

### 5.1. Part I: Results and Analysis

**Figure 7:**
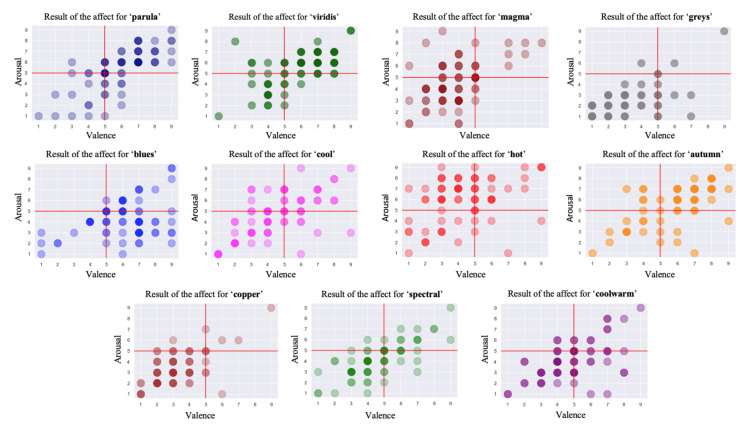
The overall result of the 11 colomaps regarding the affect evoked in the valence–arousal coordinate system.

As mentioned before, this part of the experiment was to encode the affect caused by the 11 chosen colormaps, both in valence and arousal dimensions. We determined that none of the participants had any visual impairment so their answers could be accounted as effective reference. Each affective response towards colormaps was mapped onto a numeral value by the 9-point SAM scale, allowing us to visualize the emotional results by means of the prevailing valence–arousal coordinate system. As Figure 7 shows, the 73 outcomes are characterized by each circle revealing the affect of one participant towards a certain colormap. We mirror every subfigure visually by placing a vertical and a horizontal line in the position of the value 5, which represents the neutral affect in both valence and arousal dimensions. This intuitively compartmentalizes the model into four quadrants: **positive–exciting**, **negative-exciting**, **negative-calm** and **positive-calm**. In addition, this partition coincides with the model and concepts mentioned previously.

**Figure 8:**
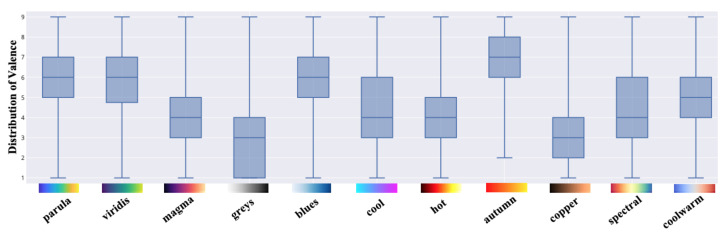
The individual distribution of the affect for the 11 colormaps in the dimension of valence.

**Figure 9:**
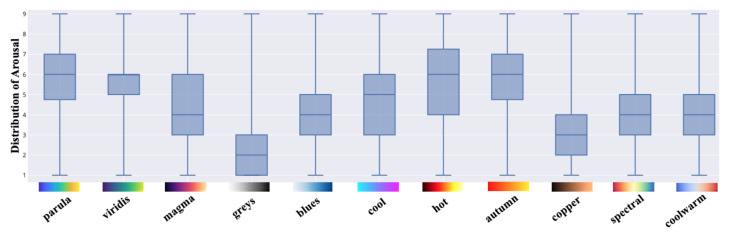
The individual distribution of the affect for the 11 colormaps in the dimension of arousal.

**Figure 10:**
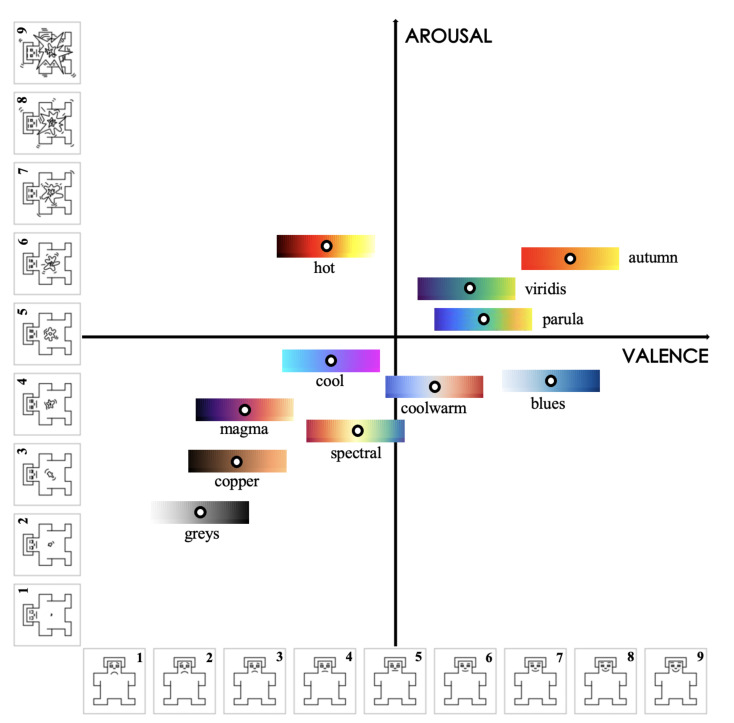
The synthetic distribution of the 11 colomaps regarding the affect evoked in the valence–arousal coordinate system. The white dots represent the exact locations of the colormaps.

Each colormap was assigned an exclusive color in this outcome visualization for better visibility and interpretability. The circles with darker chromatic concentration represent more than one answer in those positions: the darker the circle, the greater the number of the same results. Using the data collected in valence and arousal dimensions, the individual distribution of each colormap is depicted in Figure 8 and Figure 9. From these figures, we can derive a preliminary impression of which colormaps trigger affect in which quadrants of the emotional coordinate system. Thus, we can plot the synthetic distribution of the 11 colomaps of the affect evoked according to the arithmetic mean values of the two affective dimensions in one single plot (Figure 10). From our aggregated data acquired from the results and the visualized plots, it is fair to conclude that colormaps *parula*, *viridis* and *autumn* trigger emotions mostly in the **positive–exciting** quadrant. Conversely, *coolwarm*, *spectral*, *magma*, *greys*, *copper* and *cool* incite the affect mainly in the **negative-calm** quadrant. The remaining colormaps *blues* and *hot* are in the **positive-calm** and **negative-exciting** quadrants respectively.

**Figure 11:**
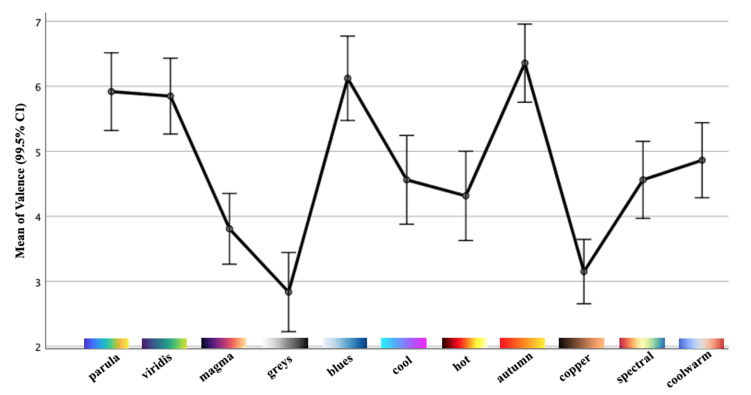
Mean values with standard errors (99.5% CI) of the valence dimension.

**Figure 12:**
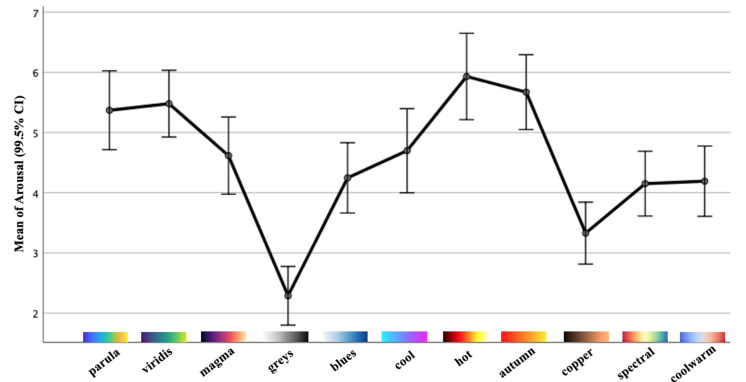
Mean values with standard errors (99.5% CI) of the arousal dimension.

To examine the effects of all 11 colormaps upon the affective responses, a one-way repeated-measures analysis of variance (rmANOVA) [37] was performed for the two dimensions included in SAM. If any single colormap or combination had statistically significant effects (*p* < 0.005), the Bonferroni-corrected post hoc tests were then performed to determine which pairs of means were significantly different. All of the analysis was performed through IBM SPSS Statistics. Table 2, along with Figure 11 and Figure 12, report the means as well as the standard deviations (SD) of valence and arousal for each colormap along with their 99.5% confidence intervals (CI). The results of the one-way rmANOVA measurement showed a significant main effect of the colormap on the affect as participants proactively reacted to the dimension of valence (*F*(10, 720) = 37.58, *p* < 0.005, $\eta_{p}^{2}$ = 0.34). Moreover, the Bonferroni post hoc tests showed statistically significant effects between every pairwise comparison of the colormaps. This indicates that the affective responses regarding each pair of colormaps with which participants reacted are statistically significant. For example, they felt a significantly more pleasant affect from the colormap *autumn* (mean = 6.36; SD = 1.78) compared to the others. Similarly, the color scheme *greys* (mean = 2.84; SD = 1.80) had the least pleasant affect in our study. Likewise, the one-way rmAVONA measurement showed that the tested colormaps had a significant main effect (*F*(10, 720) = 34.21, *p* < 0.005, $\eta_{p}^{2}$ = 0.32) in the dimension of arousal. It is noteworthy that each pairwise comparison also revealed significant effect through the Bonferroni post hoc tests. For colormap *hot* (mean = 5.93; SD = 2.12), participants reported significantly more excitement; while they sensed more calm with respect to *greys* (mean = 2.29; SD = 1.44) compared to the remaining colormaps. This evidence supports, with statistical significance, the conclusion that these colormaps can affect participants’ emotional responses.

### 5.2. Part II: Results and Analysis

**Figure 13:**
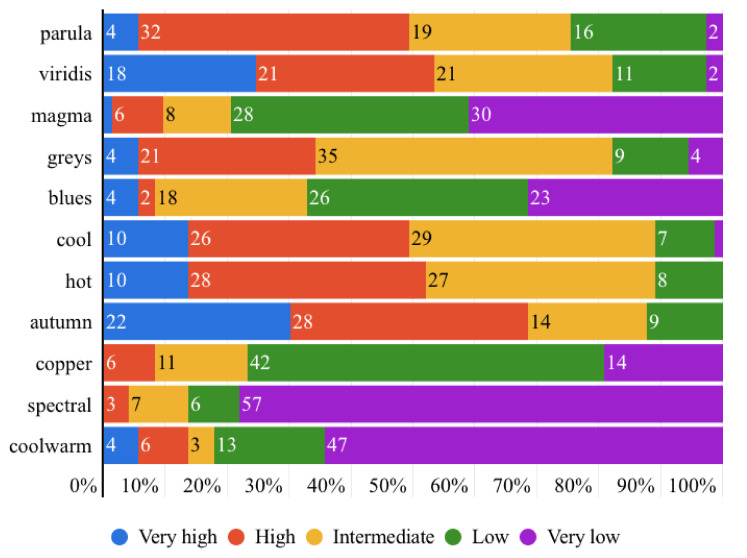
The overall accuracy rating results of the 11 colormaps by the 73 participants (rating scale: very high accuracy, high accuracy, intermediate accuracy, low accuracy and very low accuracy).

After giving the affective responses to each colormap, participants then rated the accuracy of each colormap on the same segmentation task using their own comprehension. The comprehensive statistics of the results are shown in Figure 13, which illustrates ratings of each individual colormap by the 73 participants. By excluding the neutral rating (intermediate accuracy), we intended to classify the desirable colormaps (very high accuracy and high accuracy) and undesirable colormaps (very low accuracy and low accuracy) by observing the results. Hence, it is fair to initially conclude that the colormaps *autumn*, *viridis*, *parula*, *hot* and *cool* are favored over *spectral*, *coolwarm*, *magma*, *copper* and *blues*. The further exploration of the results was conducted by a similar statistical analysis as used in Part I.

**Figure 14:**
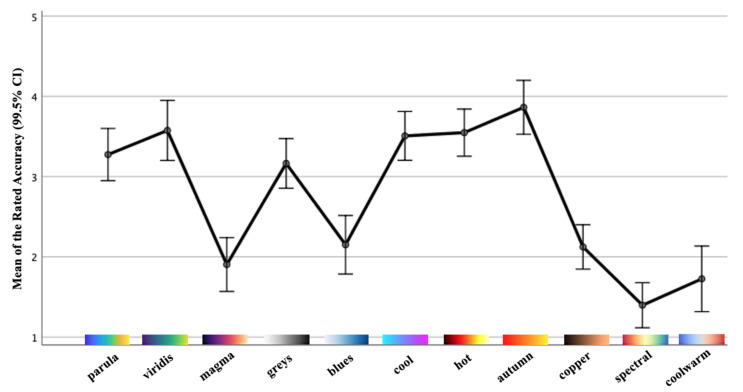
Mean values with standard errors (99.5% CI) of the colormap accuracy rated by participants.

Table 3 and Figure 14 report the means as well as the SDs of the colormap accuracy rated in crowdsourced study Part II, along with their 99.5% CIs. The results of the one-way rmANOVA measurement showed a significant main effect of the colormap on the task accuracy, which participants successfully rated (*F*(10, 720) = 69.62, *p* < 0.005, $\eta_{p}^{2}$ = 0.49). In addition, the Bonferroni post hoc tests showed that every individual colormap had a significant effect according to the pairwise comparison. For instance, participants perceived *autumn* (mean = 3.86; SD = 0.99) as significantly more accurate whilst spectral (mean = 1.40; SD = 0.83) had the least task accuracy. This shows that participants have the ability to rate the accuracy of the chosen colormaps. More specifically, these findings demonstrate that the results obtained are statistically significant. Interestingly, our results indicate that *parula*, *viridis*, *cool*, *hot* and *autumn* schemes possess superior accuracy based on participants’ visual determination, which corresponds to the initial observation in Section 3.3. However, by following the quantitative evaluation, *cool* and *hot* colormaps are practically unfavourable in our context and should be discarded. Therefore, we can confidently conclude that the color schemes *autumn*, *viridis* and *parula* are the most desirable results from the judgement by human visual comprehension. The holistic rankings of the colormaps over MWT segmentation originating from our crowdsourced study are displayed in Table 4.

### 5.3. Validation Analysis

**Figure 15:**
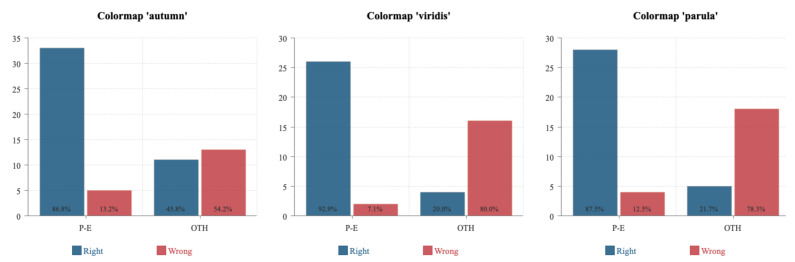
The analytic results for the three baseline colormaps *autumn*, *viridis* and *parula*. The X-axis represents the **positive–exciting** (P-E) and other quadrants (OTH) from the valence–arousal model. The Y-axis shows the number of participants who rated the designated colormap. **Right:** the number of people who rated the baseline colormap as desirable in the crowdsourced study in Part II. **Wrong:** the number of people who rated the baseline colormap as undesirable in the crowdsourced study in Part II.

From our crowdsourced study, it is noteworthy that the most accurate colormaps are *autumn*, *viridis* and *parula*, a result which corresponds to the quantitative evaluation previously made. Furthermore, these three colormaps all trigger affect in the **positive–exciting** quadrant (Figure 10 and Figure 11), which conforms to our hypothesis. Unexpectedly, we observed that the three least preferred colormaps *spectral*, *coolwarm* and *magma* are located in the **negative-calm** area. To make our findings more persuasive and robust, we carried out an additional statistical hypothesis testing of the three most preferred colormaps to verify our conclusions. We chose colormaps *autumn*, *viridis* and *parula* as baselines. We divided the results into Right (the number of people who rated the baseline colormaps as desirable in the crowdsourced study in Part II) and Wrong (the number of people who rated the baseline colormaps as undesirable in the crowdsourced study in Part II). Since we speculated that the colormaps distributed in **positive–exciting** quadrant can facilitate more accurate visual comprehension, we distributed the four quadrants of the valence–arousal coordinate into **positive–exciting** (P-E) and other quadrants (OTH) to investigate how those participants possessing affect in P-E and OTH quadrants would rate the colormaps. The overall results for these three baselines are illustrated in Figure 15. After the hypothetical verification, we found the consequential effect on *autumn* to be ($\chi^{2}$) = 12.01, α = 0.05, *p* < 0.005, *viridis* to be ($\chi^{2}$) = 26.42, α = 0.05, *p* < 0.005 and *parula* to be ($\chi^{2}$) = 24.11, α = 0.05, *p* < 0.005. From the testing outcomes, we are able to verify our hypothesis that colormaps triggering affect in the **positive–exciting** quadrant in the valence–arousal grid are able to facilitate more accurate human visual comprehension towards MWT.

## 6. Discussion

### 6.1. Insights

What do we obtain from all the results? Foremost, we proposed two core research questions regarding our study. How can various colormaps affect domain related task accuracy in the context of MWT so as to impart accurate visual comprehension? Are the colormaps triggering human affect in the **positive–exciting** quadrant in the valence–arousal grid able to facilitate more accurate visual comprehension in terms of human perception towards MWT than those from the other three quadrants? To resolve our **RQ1**, we carried out a metric-driven quantitative evaluation to judge the performance of individual colormaps based on the same segmentation task, by following the recently published work [8]. After consulting relevant literature, we selected 10 prevalent continuous colormaps (plus a default colormap, totalling 11 colormaps tested) which were capable of retaining the needed information in MWT images. The selection criterion was on the basis of categories (sequential and diverging) and color properties (single hue, binary hues and multiple hues). By means of an automatic segmentation approach, we then obtained the easily-distinguishable results which enabled us to determine the most appropriate colormaps. We found that color schemes *autumn*, *viridis* and *parula* were considered the optimal options in comprehension-based scientific analysis of MWT. In addition, we concluded that the colormaps *spectral*, *coolwarm* and *magma* were undesirable in the same context.

More critically, inspired by well-proven research on colormaps-emotion, we designed a comprehensive crowdsourced user study to address our **RQ2**, as well as to validate our hypothesis. A 73-participant involved study was formalized to verify whether the advisable colormaps concluded from the quantitative evaluation were encoded in the **positive–exciting** quadrant in our built valence–arousal model. We divided the whole study into two parts. Part I collected the affect of how participants reacted to the 11 different testing colormaps and recorded the outcomes. The SAM scale was used to encode the participants’ perceptual reactions into a measurable format. The composite results were presented in descriptive tables and figures, which showcase the entire displacement of the 11 tested colormaps in the valence–arousal coordinate system. The rmANOVA measurement tool solidly confirmed that the acquired results were statistically significant. In Part II, our complementary study investigated the ratings by the participants of the segmentation accuracy of each colormap. We used the well-known Likert scale to record the data. From the outcome presentation, we demonstrated that our results had a statistically significant effect. Through integrating the two parts, we expectedly found that the most desirable colormaps *autumn*, *viridis* and *parula* indeed trigger the human affect in the **positive–exciting** quadrant which conforms with our hypothesis. To robustly justify our findings, we set up an additional statistical hypothetical test that then effectively further proved the correctness of our results. Moreover, by referring to the well-known valence–arousal model, we have shown its accessibility and suitability in our affective design study.

Why did we obtain such results? Colormaps can result in different domain related task accuracy, especially in our comprehension oriented contexts. The answer of **RQ1** was obtained by an exhaustive quantitative evaluation across three renowned indexes, which led us to find the objective color schemes. Moreover, why can we successfully verify our hypothesis regarding **RQ2**? On one hand, the chosen three best colormaps are intrinsically suitable in supporting task accuracy in industrial tomography. On the other hand, the human operator is a critical component in the context. It is vital for domain related users to understand the tomographic images by their visual comprehension. Human affect from proper colormap selection has the capacity to influence the accuracy-critical decision-making. In line with [14], the chromatic properties of colormaps are more intensive in positive and exciting dimensions and are milder in negative and calm dimensions. The three desirable colormaps intensively trigger energetic affect like joy and happiness, which can effectively underpin human decision-making within cognitive tasks. On the contrary, it is therefore reasonable to deduce that the three undesirable colormaps having passive affect like sadness may negatively influence human cognitive abilities.

Beyond the fundamental research output, the design implications of our work has high transferability. We chose the MWT as a representative object along with a specific microwave foam drying process. However, our findings are equally applicable to other modalities in industrial tomography due to the high interoperability, for example, industrial batch crystallization using Electrical Resistance Tomography (ERT) or inline fluid separation using Electrical Capacitance Tomography (ECT).

Our goal is to seek a breakthrough in affective colormap design research. Color coding in various application settings and emotional color visualization have been well researched over the past decades. The current work has investigated either benchmarking colormaps by various tasks [18] or assessing the colormaps by hands-on user testing such as crowdsoucing [20]. We, however, have especially incorporated the conventional task-based design strategy along with the emerging crowdsourced user study to investigate the affective colormap design in the domain of industrial tomography. In response to our two research questions, including a preliminary hypothesis, a quantitative evaluation followed by a thorough user study was established for exploration. After rigorous analysis in terms of both results and statistics, we favorably validated our prior assumption.

### 6.2. Limitations

While these results show a satisfactory performance for using affective colormap design to advance human visual comprehension in the domain of industrial tomography, we acknowledge several existing limitations. First, we investigated 11 different colormaps in terms of several selection strategies, in which categories (sequential and diverging) and color properties (hues and lightness) were considered. However, the design strategy of additional colormaps could be enriched by adding more classes of colormaps that might then give similar or more outstanding performance in our context, for instance, other breeds of colormaps like cyclic and qualitative colormaps. Furthermore, another important property—color chroma—should be considered in choosing appropriate testing objects.

Second, we chose continuous colormaps simply because of their ability to retain complete information of MWT as well as respecting the default setting in MATLAB. However, the scope could be extended by inspecting discrete colormaps. Since we evaluated the color schemes through an image segmentation task, it could be promising to introduce a suitable number of discrete colormaps into our study. Thirdly, the number of tested objects (both of the number of colormaps and the participants involved in the user study) is not considerably sufficient. Although we have gained new, conclusive insights, a larger number of colormaps, as well as a larger scale of user study could be studied in the future.

Some factors influencing the crowdsourced study could not be controlled. For instance, we recruited participants from a range of domains while the working environment of each participant could not be controlled. Participants most likely were subject to different screen resolution, lighting and other environmental factors. A unified and subjective working environment could have ensured more robust results.

Finally, even though our result powerfully supports the potential to leverage affective colormaps design in boosting visual comprehension in the domain of MWT, a representative type of industrial tomography, it cannot be firmly concluded that our results can be generalized in the context of each breed of tomography. This is due to the sophisticated categories of tomography. For example, computed tomography (CT), which is pervasively used in medical scenarios, is completely different from industrial tomography. Whether our conclusions could be used in CT needs to be investigated.

## 7. Conclusions

In this paper, we aimed to investigate how to support accurate visual comprehension in industrial tomography. The non-destructive MWT was selected as the research context. A quantitative evaluation of our work showed that different colormaps can influence the task accuracy in MWT related analytics and that schemes *autumn*, *virids* and *parula* can provide the best performance. In our systematic crowdsourced study, we verified our hypothesis that the colormaps triggering affect in the **positive–exciting** quadrant in the valence–arousal model are able to facilitate more precise visual comprehension in MWT than the other three quadrants. Interestingly, we also discovered the converse-finding that colormaps resulting in affect in the **negative-calm** quadrant are undesirable. Therefore, we propose a synthetic design guideline for future practitioners to select colormaps boosting accurate visual comprehension in the context of MWT.

- **Guideline-1:** For comprehension-based segmentation scientific analysis for MWT, we recommend the colormaps *autumn*, *viridis* and *parula* as the most suitable color schemes. For the same context of use, we advise against *spectral*, *coolwarm* and *magma* schemes.
- **Guideline-2:** For comprehension-based segmentation scientific analysis for MWT, we recommend the colormaps triggering affect in the **positive–exciting** quadrant in the valence–arousal emotion model as the most suitable color schemes to facilitate visual comprehension. Conversely, we advise against those color schemes which incite affect in the **negative-calm** quadrant.

Our research is novel and has various possibilities in relevant future studies. We admit that we have not yet tested a wider range of representative colormaps. We chose continuous colormaps because they reveal the most useful information in MWT. We believe that discrete colormaps should be examined in future work. In addition, other categories of colormaps, like cyclic and qualitative colormaps, should also be investigated. A much larger number of sample images (this study used eight) could make our study more generic and persuasive. Moreover, there is room to improve our crowdsourced study, for instance, by involving more participants from a wider range of backgrounds. Since human perception differs individually [38] for colors and images, results acquired and analyzed upon a more substantial number and range of participants would be more reliable. Meanwhile, comparing and analyzing different results derived from different groups (experts and non-experts) may lead to other interesting findings. Last but not least, we have successfully proven that some designated colormaps are more suitable than others in the domain of industrial tomography. However, it is critical to examine our findings for other types of tomography in future work. Overall, the aim of our research was to demonstrate the potential of colormap design to support novel industrial tomography segmentation procedures. Our results have proven that this potential is worthy of future investigation.

## Figures and Tables

**Table 1:** The specification of the user study carried out, including the stimuli and the anticipated results.

	The Stimuli Presented to Participants	The Anticipated Results from Participants
***The user study***		
**Part I**	The 11 colormaps used in the study	Affective responses for each colormap
**Part II**	The 11 colormaps used in the study	Comprehension ability in rating
		the task accuracy of each colormap

**Table 2:** Summary of the mean values and standard deviations of the affective responses obtained in crowdsourced study Part I with 99.5% CI.

	Parula	Viridis	Magma	Greys	Blues	Cool	Hot	Autumn	Copper	Spectral	Coolwarm
***Valence***			
**Mean**	5.92	5.85	3.81	2.84	6.12	4.56	4.32	6.36	3.15	4.56	4.86
**SD**	1.762	1.721	1.604	1.795	1.914	2.014	2.027	1.775	1.459	1.748	1.702
***Arousal***			
**Mean**	5.37	5.48	4.62	2.29	4.25	4.70	5.93	5.67	3.33	4.15	4.19
**SD**	1.933	1.634	1.890	1.438	1.722	2.059	2.117	1.834	1.519	1.587	1.721

**Table 3:** Summary of the mean values and standard deviations of the colormap accuracy rated in crowdsourced study Part II with 99.5% CI.

	Parula	Viridis	Magma	Greys	Blues	Cool	Hot	Autumn	Copper	Spectral	Coolwarm
***Accuracy***			
**Mean**	3.27	3.58	1.90	3.16	2.15	3.51	3.55	3.86	2.12	1.40	1.73
**SD**	0.961	1.105	0.988	0.913	1.076	0.899	0.867	0.990	0.816	0.829	1.205

**Table 4:** The holistic accuracy rankings (high to low) of the 11 colormaps obtained from study part 2.

Ranks	1	2	3	4	5	6	7	8	9	10	11
Colormaps	autumn	viridis	parula	hot	cool	greys	blues	copper	magma	coolwarm	spectral

## Data Availability

The data presented in this study are available on request from the corresponding authors.
